# NETs promote invasive behavior of fibroblast-like synoviocytes through GPIbα in rheumatoid arthritis

**DOI:** 10.3389/fimmu.2025.1667319

**Published:** 2025-11-21

**Authors:** Yongqiang Zhang, Yingyi Wu, Hui Yang, Xuanqi Liu, Bing Pan, Congzhu Ding, Yue Sun, Lingyun Sun

**Affiliations:** 1 Department of Rheumatology and Immunology, Nanjing Drum Tower Hospital, Clinical College of Nanjing University of Chinese Medicine, Nanjing, China; 2 Department of Rheumatology and Immunology, Huaihe Hospital, Henan University, Kaifeng, China; 3 Department of Rheumatology and Immunology, China Pharmaceutical University Nanjing Drum Tower Hospital, Nanjing, China; 4 Department of Rheumatology and Immunology, Nanjing Drum Tower Hospital Clinical College of Nanjing Medical University, Nanjing, China; 5 Department of Rheumatology and Immunology, Nanjing Drum Tower Hospital, Affiliated Hospital of Medical School, Nanjing University, Nanjing, China

**Keywords:** rheumatoid arthritis (RA), fibroblast-like synoviocytes (FLS), neutrophil extracellular traps (NETs), glycoprotein Ib alpha (GPIbα), synovitis

## Abstract

**Objective:**

Neutrophil extracellular traps (NETs) have been implicated in rheumatoid arthritis (RA) pathogenesis, yet their effects on fibroblast-like synoviocytes (FLS) remain unclear. This study aims to investigate the role of NETs in RA FLS migration, proliferation, and invasion, as well as the underlying molecular mechanisms.

**Methods:**

NETs formation was assessed in neutrophils isolated from RA patients and healthy controls (HC) using immunofluorescence staining. RA FLS were stimulated by RA or HC derived NETs, and migration was evaluated via wound healing assays. RNA sequencing (RNA-seq) identified differentially expressed genes in FLS, validated by qPCR. The expression and localization of glycoprotein Ib alpha (GPIbα) in RA synovium were examined by immunohistochemistry. GPIbα was knocked down in FLS to assess its role in proliferation and migration. A collagen-induced arthritis (CIA) model was used to study the effect of NETs inhibition on RA progression.

**Results:**

RA neutrophils produced more NETs than HC neutrophils. RA NETs enhanced FLS migration and proliferation, and RNA-seq revealed upregulation of *GP1BA*, which was confirmed by qPCR. GPIbα expression was elevated in RA synovium. *GP1BA* knockdown suppressed RA NETs-induced FLS proliferation and migration. In CIA mice, inhibiting NETs formation decreased GPIbα expression, limited FLS invasiveness, and attenuated RA progression.

**Conclusion:**

Our findings reveal that NETs promote RA progression by inducing FLS proliferation and migration through GPIbα. Consequently, targeting NETs formation or GPIbα represents a promising therapeutic strategy to mitigate RA.

## Introduction

Rheumatoid arthritis (RA) is a chronic, systemic autoimmune disease that primarily affects synovial joint, leading to synovitis and joint destruction (1). Over time, this pathological process can result in joint deformity and functional impairment. Although the precise etiology of RA remains incompletely understood, it is widely accepted that its onset is closely associated with genetic predisposition, environmental factors, and immune system dysregulation (2–4). Current therapeutic strategies for RA include nonsteroidal anti-inflammatory drugs (NSAIDs), disease-modifying antirheumatic drugs (DMARDs), and biologic agents (5). However, these treatments exhibit various limitations in terms of efficacy, cost, and adverse effects, underscoring the critical need to identify novel therapeutic targets for optimizing RA management.

One of the hallmark pathological features of RA is synovial tissue remodeling, which is characterized by aberrant proliferation of fibroblast-like synoviocytes (FLS) (6, 7). Within the RA synovium, activated FLS secrete proinflammatory cytokines, such as interleukin-6 (IL-6), as well as matrix metalloproteinases (MMPs), thereby exacerbating synovial inflammation and accelerating cartilage degradation (8, 9). Studies have demonstrated a marked increase in FLS numbers within RA synovial tissue, leading to structural remodeling of the synovial lining and ultimately contributing to the formation of an aggressive hyperplastic tissue mass known as pannus (10, 11). Consequently, targeting the abnormal proliferation and activation of RA FLS represents a promising therapeutic strategy for mitigating disease progression and joint damage in RA (12).

Within the RA joint microenvironment, in addition to FLS, neutrophils also play a critical role in synovial inflammation and tissue destruction. While neutrophils are essential for antimicrobial immunity and acute innate immune responses, their aberrant recruitment and excessive activation can contribute to immune-mediated tissue damage (13, 14). During the early stages of RA or acute disease flares, neutrophils are the most abundant immune cells in the synovial fluid (15–17). Previous studies have demonstrated that activated neutrophils release cytotoxic products, such as degradative enzymes and reactive oxygen species (ROS), into the synovial fluid and pannus, thereby exacerbating local inflammation and accelerating cartilage destruction (17, 18). Moreover, accumulating evidence suggests that neutrophils may have more complex functional roles in RA, beyond their traditional inflammatory activity. In particular, the formation of neutrophil extracellular traps (NETs) has emerged as a key research focus in RA pathogenesis (19, 20). NETs are web-like extracellular structures released by neutrophils in response to specific stimuli, composed primarily of extracellular chromatin decorated with histone modifications and various granule-derived proteins (21). The process of NETs formation, known as NETosis, represents a unique form of programmed cell death, distinct from apoptosis and necrosis, and has been recognized as an integral component of the innate immune response (22). Under physiological conditions, NETs serve to limit pathogen dissemination and enhance host immune defense (23, 24). Nevertheless, under certain pathological conditions, NETs may accumulate excessively, leading to chronic inflammation and tissue damage (23–26).

NETs have been increasingly implicated in the pathogenesis of autoimmune diseases. In systemic lupus erythematosus (SLE), impaired clearance of NETs and the exposure of modified autoantigens can break immune tolerance and promote autoantibody production (27). In antineutrophil cytoplasmic antibody (ANCA)-associated vasculitis, NETs have been shown to contribute to vascular endothelial injury and serve as a source of autoantigens (28). Similarly, in psoriasis, NETs formation is enhanced and may amplify inflammation through the activation of plasmacytoid dendritic cells and T cells (29). These findings across different autoimmune contexts underscore the broad significance of NETs as a key mediator of autoimmunity and highlight the need to elucidate their disease-specific mechanisms.

Recent studies have confirmed that NETs play a crucial role in RA-associated bone erosion. For example, it has been reported that NETs promote osteoclast differentiation, thereby directly contributing to bone destruction in RA (30, 31). However, the specific impact of NETs on FLS function and their mechanistic role in RA progression remain largely unexplored. In this study, we investigated the direct role of RA NETs on RA FLS proliferation, migration, and invasive capacity, and further explored the underlying molecular mechanisms. Our findings not only provide new insights into the role of NETs in RA pathogenesis but also offer a potential foundation for developing novel targeted therapeutic strategies for RA intervention.

## Materials and methods

### Patients and clinical sample collection

To investigate the role of NETs in RA, we first obtained clinical samples from well-characterized patient cohorts. All experimental protocols were approved by the Ethics Committee of Nanjing Drum Tower Hospital (*reference number* 2021-544-01). Written informed consent was obtained from all patients. RA patients were recruited from the Department of Rheumatology and Immunology at Nanjing Drum Tower Hospital and fulfilled either the 2010 American College of Rheumatology (ACR)/European League Against Rheumatism (EULAR) classification criteria or the 1987 ACR classification criteria (32, 33). Inclusion criteria required patients to be aged 18 years or older. Exclusion criteria included concomitant rheumatic diseases, severe infections, malignancy, pregnancy, and lactation. Peripheral blood was collected from RA patients and age-and-sex matched healthy controls (HC). Human synovial tissues were obtained from RA patients and age-and-sex matched osteoarthritis (OA) patients undergoing knee replacement surgery at our institution.

### Animal model of arthritis

To extend our *in vitro* findings to an *in vivo* setting, we utilized a murine model of inflammatory arthritis. Eight-week male DBA1 mice were purchased from Chares River. All animal experiments were performed under an institutionally approved protocol for the use of animal research. Collagen-induced arthritis (CIA) was induced as previously described (34). Briefly, bovine type II collagen (Chondrex) was dissolved at 4 °C overnight in acetic acid and emulsified with an equal volume of complete or incomplete Freund’s adjuvant (Chondrex). Mice were immunized with 100μg of type II collagen by intradermal injection at the base of the tail on day 1 and day 21. To assess the specific contribution of NETs to disease progression, we administered peptidyl arginine deiminase 4 inhibitor GSK 484 (Cayman) after disease onset (day 28), when arthritis had become established (arthritis score ≥2). CIA mice received daily intraperitoneal injections of either 200ul saline or GSK484 (4 mg/kg) for 14 days. Healthy DBA1 mice served as negative control. On day 42, mice were euthanized, and blood, bone marrow and ankle joints were collected for subsequent studies. Arthritis scoring was performed twice weekly according to the previous protocol (35).

### Isolation of neutrophils and induction of NETs

To generate NETs for subsequent functional assays, neutrophils were isolated from peripheral blood of RA patients or HC using Polymorphprep (Axi-Shield) following the manufacturer’s instructions (36). Neutrophils were suspended in RPMI-1640 (Gibco) containing 3% fetal bovine serum (FBS) and seeded into 6-well plates (1×10^6^cells/well). After incubation at 37 °C and 5% CO_2_ for 4h under non-interventional conditions, NETs were digested with 10U/mL of micrococcal nuclease (Thermo Fisher) for 15min at 37 °C. Supernatants containing NETs fragments were collected and centrifuged at 1,000g for 10min to remove cellular debris. Separated NETs were transferred to fresh tubes and stored at -80 °C until further use.

### Detection of NETs formation

To confirm and quantify NETs formation, we employed both immunofluorescence microscopy and flow cytometry. Neutrophils isolated from RA or HC peripheral blood were cultured into 24-well plates (1×10^5^cells/well) and incubated undisturbed at 37 °C and 5% CO_2_ for 4h. Cells were subsequently fixed with 4% paraformaldehyde for 15min, followed by blocking with 2% bovine serum albumin (BSA) for 1h. Cells were incubated overnight at 4 °C with the following antibodies: anti-myeloperoxidase (MPO) mAb (Thermo Fisher) or anti-neutrophil elastase (NE) mAb (Thermo Fisher). DNA was stained using 4′,6-diamidino-2-phenylindole (DAPI) (Beyotime Biotechnology). Neutrophils isolated from mouse blood or bone marrow were cultured as above, and then were stained with anti-MPO mAb (Thermo Fisher). NETs were observed by a confocal fluorescence microscope (Olympus), and two independent individuals manually quantified neutrophils and NETs. For flow cytometric analysis, RA or HC derived neutrophils (8×10^3^ cells/well) were seeded into 96-well plates and incubated in a humidified incubator at 37 °C with 5% CO_2_ for 4h, without any intervention. Following incubation, cells were stained with SYTOX Green, a membrane-impermeable DNA-binding dye, in accordance with the manufacturer’s instructions (Invitrogen). Cellular debris was removed by filtration through a mesh, and the remaining cells were subsequently analyzed using the Fortessa flow cytometer (BD Biosciences).

### Isolation of fibroblast-like synoviocytes

Synovial tissues from RA patients were minced into small pieces and digested in 0.2% of type I collagen (Sigma) dissolved in DMEM (Gibco) for 10h at 37°C in 5% CO_2_. Digestion was terminated by adding DMEM with 15% FBS. The cell suspension was filtered through a 100μm cell strainer (Falcon) and resuspended in DMEM with 15% FBS. Cells were cultured for 24 hours to allow adherence, after which the medium was changed to remove non-adherent cells, and the FBS concentration was reduced to 10%. The medium was changed every 2 or 3 days and the cells were passaged at 80% confluence. FLS at passage 3–6 were used for subsequent experiments.

### Proliferation and migration assays

To determine whether NETs influence FLS proliferation, we performed CCK8 assays. RA FLS (1×10^4^ cells/well) were seeded in 96-well plates overnight. Cells were then cultured with RA or HC NETs at various concentrations of 0, 5, 50, and 500ug/ml for 24h, 48h or 72h. After incubation, 10μl of CCK8 reagent (Biosharp) was added to each well and incubated at 37 °C for 2h. The absorbance at 450nm was measured to calculate cell viability. To assess the effect of NETs on FLS migration, we conducted wound healing assays. RA FLS were seeded in 6-well plates at a density of 1×10^6^ cells/well. Once confluent, a uniform scratch was created using a pipette tip, followed by washing with PBS to remove detached cells. DMEM supplemented with 5% FBS and either RA or HC NETs (50μg/mL), or control medium without NETs, was added to the wells. Photographs of the scratch were taken at baseline (0h) and 24h post-treatment. Scratch distance and area were measured using image analysis software. Each condition was tested in three independent experiments.

### RNA sequencing and bioinformatic analysis

To explore the molecular mechanisms underlying NETs’ effects on FLS, we performed transcriptome analysis. RA FLS were treated with RA or HC NETs (50ug/ml) for 24h, with untreated FLS serving as controls. Total RNA was isolated and detected for quality control. Library construction, quality inspection, and sequencing were performed by Novogene Bioinformatics Technology Co., Ltd. using the Illumina NovaSeq 6000 system. Differential expression analysis (three biological replicates per condition) was performed using the DESeq2 package. Transcripts with *P* values of <0.05 and absolute values of log2 (fold change) >1 were assigned as differentially expressed transcripts.

### Reverse transcription quantitative PCR

To validate key findings from RNA sequencing, we performed RT-qPCR. Total RNA was extracted from RA FLS or mouse synovium tissues with TRIzol reagent (Invitrogen). cDNA was synthesized from 1μg RNA using RT Mix II (Vazyme Biotech). Real-time PCR was performed using SYBR Green Master Mix (Vazyme Biotech) with the following primers: *hGAPDH*: 5′-GCACCGTCAAGGCTGAGAAC-3′ (forward), 5′-GCACCGTCAAGGCTGAGAAC-3′(reverse); *hGP1BA*: 5′-ACCATCCTGGTGTCTGCCACAA-3′ (forward), 5′-ACGGAGCTTTGGTGGCTGATCA-3′ (reverse); *hZNF564*: 5′-ATGGGAAGACCAGAGCATTGAAG-3′ (forward), 5′-TGACTGAAGGCTTCTCCACATTG-3′ (reverse); *hADGRG2*: 5′-CTGGTCAGACAATGGCTGCTCT-3′(forward), 5′-CAGAGCCATCATTTGAGCAGGC-3′(reverse); *mGAPDH*: 5′-CATCACTGCCACCCAGAAGACTG-3′(forward), 5′-ATGCCAGTGAGCTTCCCGTTCAG-3′(reverse); *mGP1BA*: 5′-GATGTGCCAACTTGGACAATGCG-3′(forward), 5′-CTTGACCTCAGTTCTTGTGGCAG-3′ (reverse). The StepOnePlus system (Applied Biosystems) was used for real-time PCR. Data were analyzed using the 2^-ΔΔCt^ method, normalized to *GAPHD*.

### Small interfering RNA transfection

To establish a causal relationship between identified genes and NETs’ effects, we performed gene knockdown experiments. Commercially available siRNA targeting *GP1BA* and control reagents (siNC) were purchased from Invitrogen and transduced at a concentration of 0.2μM using Opti-MEM and Lipofectamine2000. After transduction, RA FLS were stimulated with RA NETs (50ug/ml) for 24h, followed by proliferation and migration assays. RA FLS without NETs stimulation were used as blank control.

### Western blot

Proteins were extracted from human or mouse synovium tissues with RIPA buffer containing phosphatase and protease inhibitors and were separated on 10% acrylamide/bis-SDS gels (Epizyme) and transferred onto polyvinylidene fluoride membranes (Millipore). After blocking with 5% BSA (Sinopharm) for 1h at room temperature, membranes were incubated with primary antibodies against GAPDH (CST, 1:2000) or GPIbα (Proteintech, 1:2000) overnight at 4°C. Membranes were then incubated in secondary antibodies (CST, 1:4000) for 1h at room temperature. After repeated washing, bands were scanned by the Tanon-5200 chemiluminescent imaging system and semi-quantified with ImageJ Software.

### Histological and immunohistochemical analysis

To examine pathological changes and protein expression *in situ*, we performed histological staining. Sections of human synovium or mouse ankle joints were deparaffinized and hydrated with gradient alcohol. For hematoxylin and eosin (H&E) staining, sections were stained with H&E, dehydrated, and mounted with neutral gum. Histopathological changes were observed and recorded using an optical microscope. For immunohistochemistry, tissues were fixed with 4% paraformaldehyde overnight, dehydrated through graded ethanol, cleared, and embedded in paraffin. Sections were stained with antibodies against GPIbα, ZNF564 or ADGRG2 (all from Proteintech, 1:1000). Staining results were observed under an optical microscope.

### Immunofluorescence staining

To visualize protein localization and co-expression, we performed immunofluorescence staining on paraffin-embedded mouse ankle joints. Sections were deparaffinized, antigen-repaired using sodium citrate buffer, and blocked with immunofluorescence blocking buffer (CST). Sections were incubated overnight at 4°C with primary antibodies against GPIbα, Vimentin and Ki-67 (all from Proteintech). After PBS washing, secondary antibodies (Jackson ImmunoResearch) were added. Sections were incubated at 37°C for 50min in the dark, counterstained with DAPI for 10min at room temperature. and sealed with anti-fluorescence quenching tablets. Images were acquired using a confocal microscope (Nikon).

### Statistical analysis

Graphpad Prism 8.0 software was used for statistical analysis. Data were expressed as mean ± standard deviation (SD). Paired data from two groups with non-normal distribution were analyzed by the non-parametric Wilcoxon signed-rank test. Multiple group comparisons of normally distributed data were conducted using an analysis of variance (ANOVA). Paired data from multiple groups with non-normal distribution were compared by the Friedman test. *p* < 0.05 was considered statistically significant. *p*-values: ns, *p*≥0.05; *, *p* < 0.05; **, *p* < 0.01; ***, *p* < 0.001; ****, *p* < 0.0001.

## Results

### NETs levels were increased in RA patients

**Figure 1:**
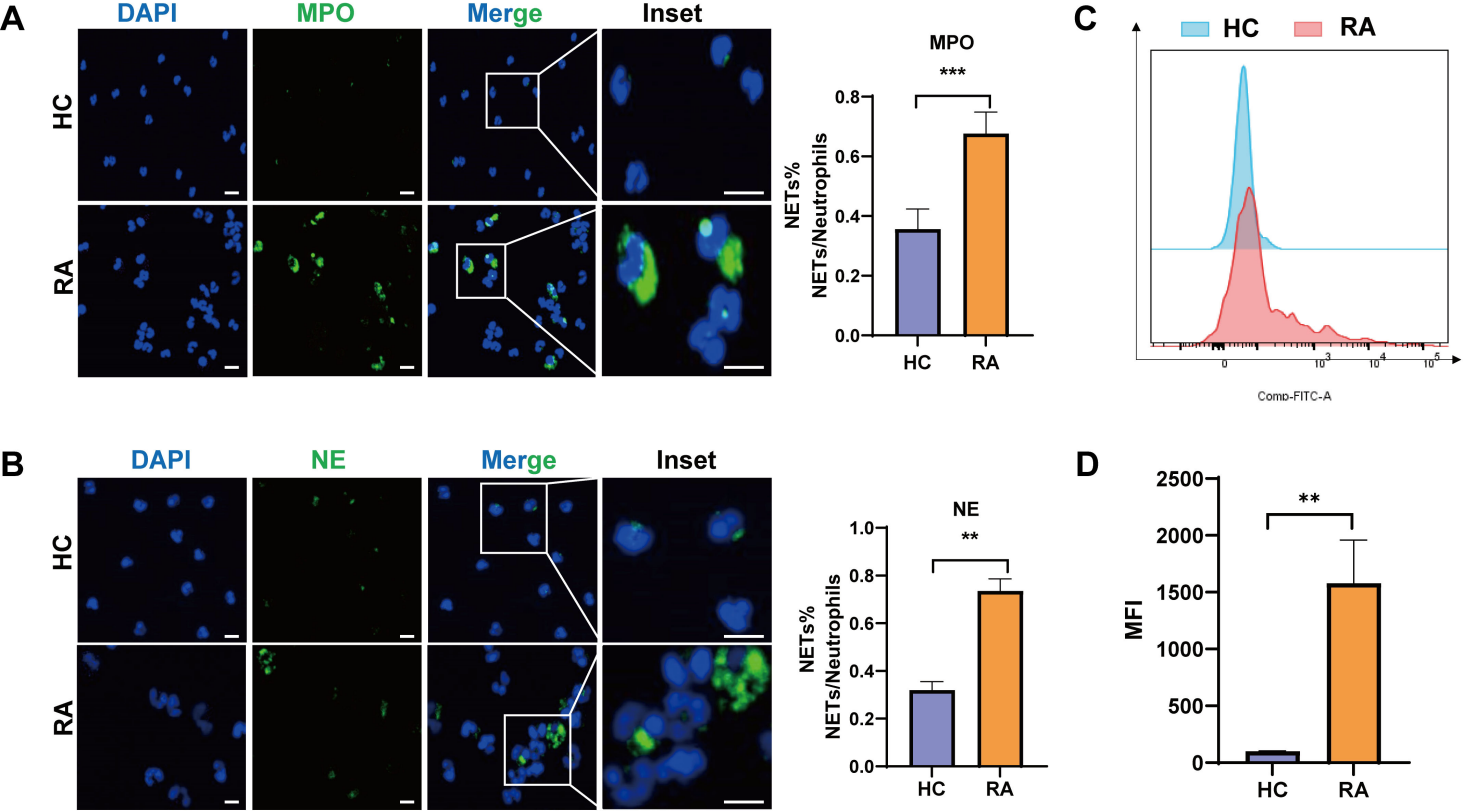
NETs levels were increased in RA patients. **(A, B)** Representative immunofluorescence images showing the release of myeloperoxidase (MPO, green) and neutrophil elastase (NE, green) from peripheral polymorphonuclear neutrophils of healthy controls (HC) or RA patients. DNA was counterstained with DAPI (blue). Scale bars represent 100μm for the main images and 100μm for the inset images. Quantifications of the percentage of NETs in neutrophils from HC or RA patients are shown in the right panels. **(C)** Representative flow cytometry profiles of SYTOX Green labeled live neutrophils isolated from HC and RA peripheral blood, showing the level of NETs formation. **(D)** Quantifications for the mean fluorescence intensity (MFI) of SYTOX Green in neutrophils from HC and RA peripheral blood. **P < 0.01, ***P < 0.001.

To determine the levels of NETs formation in RA patients, neutrophils isolated from the peripheral blood of RA patients and healthy controls (HC) were stained with myeloperoxidase (MPO) and neutrophil elastase (NE). NETs were identified by the extracellular co-localization of MPO or NE with DNA, as visualized by DAPI staining (37). The results demonstrated that neutrophils from RA patients produced significantly more NETs than those from HC, consistent with previous studies (38) (**Figures 1A, B**). Furthermore, flow cytometry using SYTOX Green, a membrane-impermeable DNA-binding dye, revealed a higher mean fluorescence intensity (MFI) in RA neutrophils, further confirming enhanced NETs formation in RA patients. (**Figures 1C, D**).

### RA NETs promoted RA FLS proliferation and migration

**Figure 2:**
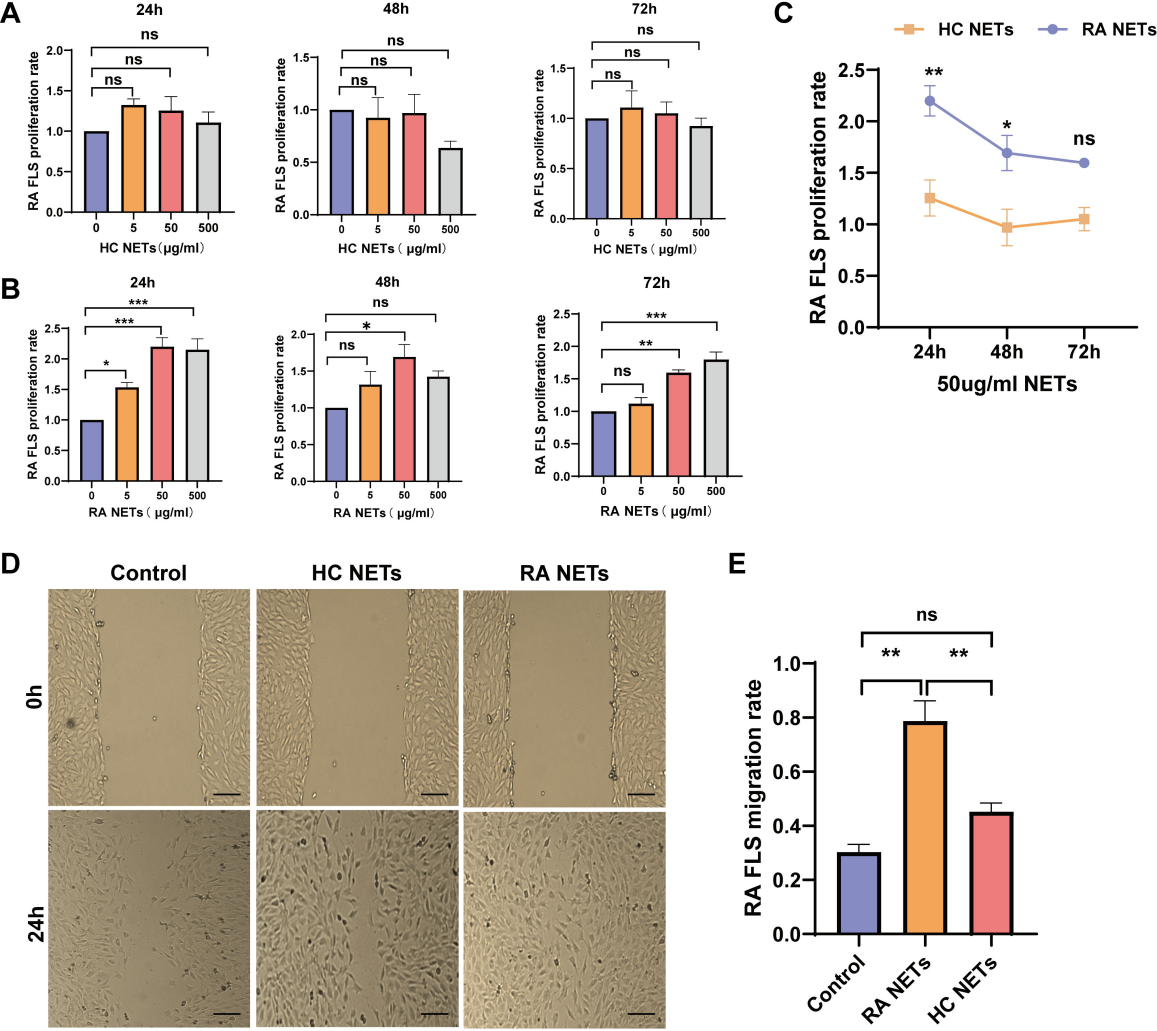
RA NETs promoted RA FLS proliferation and migration. **(A-C)** RA FLS were stimulated with different concentrations of HC NETs or RA NETs for 24 h, 48 h or 72 h, and cell proliferation was measured by CCK8 assay. **(D, E)** The effect of HC or RA NETs (50μg/ml) on RA FLS migration was evaluated using a scratch wound healing assay. Representative images of the migration assay are shown in **(D)** (scale bar: 100μm). Quantification of migration rates is shown in **(E)**. *P < 0.05, **P < 0.01, ***P < 0.001; ns, not significant.

To assess the effects of NETs on RA FLS proliferation, RA FLS were treated with varying concentrations of RA NETs (5, 50, or 500 µg/ml) for 24h, 48h and 72h, followed by CCK8 assay to evaluate cell proliferation. RA NETs at 50 and 500 µg/ml significantly promoted FLS proliferation, whereas 5 µg/mL RA NETs and all concentrations of HC NETs showed no notable effect (**Figures 2A, B**). Based on these results, 50 µg/ml RA NETs for 24h was selected for subsequent experiments (**Figure 2C**). In wound healing assays, RA NETs significantly enhanced FLS migration compared to HC NETs (**Figures 2D, E**). Collectively, these findings suggest that RA NETs directly promote RA FLS proliferation and migration.

### GP1BA: A target gene upregulated in RA FLS induced by RA NETs

**Figure 3:**
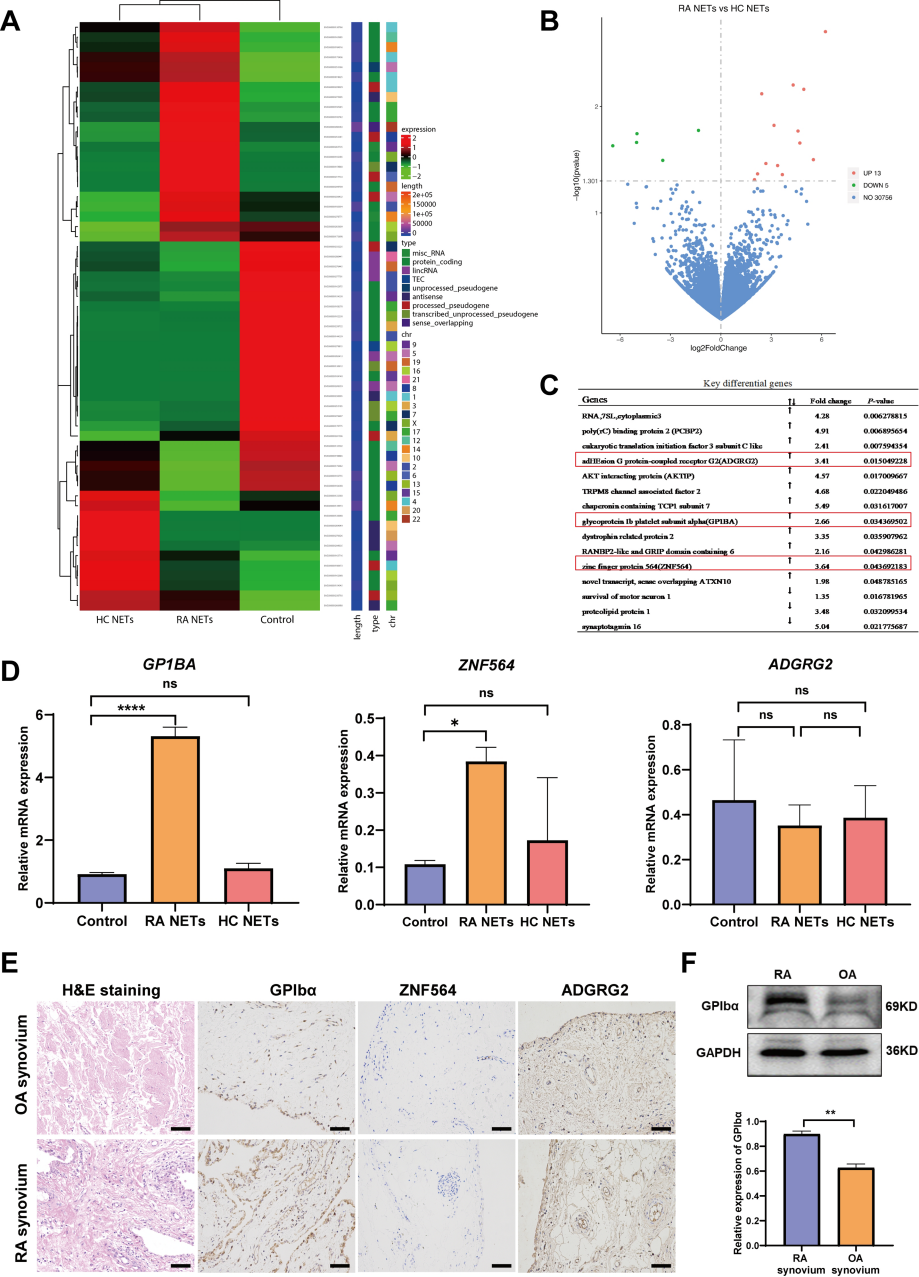
*GP1BA*: A target gene upregulated in RA FLS induced by RA NETs. **(A)** Clustering heatmap of differentially expressed genes following stimulation with HC NETs, RA NETs or control medium. **(B)** Volcano plot of gene expression changes in RA FLS upon RA NETs stimulation compared to HC NETs. **(C)** RNA sequencing analysis confirming upregulation of *GP1BA, ZNF564 and ADGRG2* in RA FLS stimulated with RA NETs. **(D)** RT-qPCR analysis of *GP1BA, ZNF564 and ADGRG2* expression in RA FLS following 24h of RA NETs or HC NETs stimulation. **(E)** Immunohistochemical staining showing GPIbα expression in RA and OA synovium (brown staining indicates positive expression, scale bar: 100μm). **(F)** Western blot analysis of GPIbα expression in RA and OA synovium, with relative quantification shown below. *P < 0.05, **P < 0.01, ***P < 0.001; ns, not significant.

To further elucidate the molecular mechanisms underlying RA NETs-induced FLS invasion, RNA sequencing was performed on RA FLS treated with RA NETs, HC NETs, or control medium (**Figure 3A**). A total of 13 upregulated and 5 downregulated genes were identified in RA FLS treated with RA NETs (**Figure 3B**). Based on differential gene expression analysis and relevant literature review, three candidate genes—*GP1BA*, *ZNF564*, and *ADGRG2*— were selected for further validation due to their potential roles in FLS proliferation and migration (39–41) (**Figure 3C**). RT-qPCR showed that only *GP1BA* expression was significantly increased upon RA NETs stimulation, whereas *ZNF564* and *ADGRG2* showed minor or no changes (**Figure 3D**). Immunohistochemistry further demonstrated that GPIbα expression was significantly elevated in RA synovial tissues compared to OA patients, whereas ZNF564 and ADGRG2 showed no notable differences between the two groups (**Figure 3E**). Western blot analysis also confirmed upregulated GPIbα expression in RA synovial tissues (**Figure 3F**). Altogether, our findings suggest that NETs may enhance FLS invasiveness through upregulation of GPIbα.

### RA NETs promoted RA FLS proliferation and migration via GP1BA

**Figure 4:**
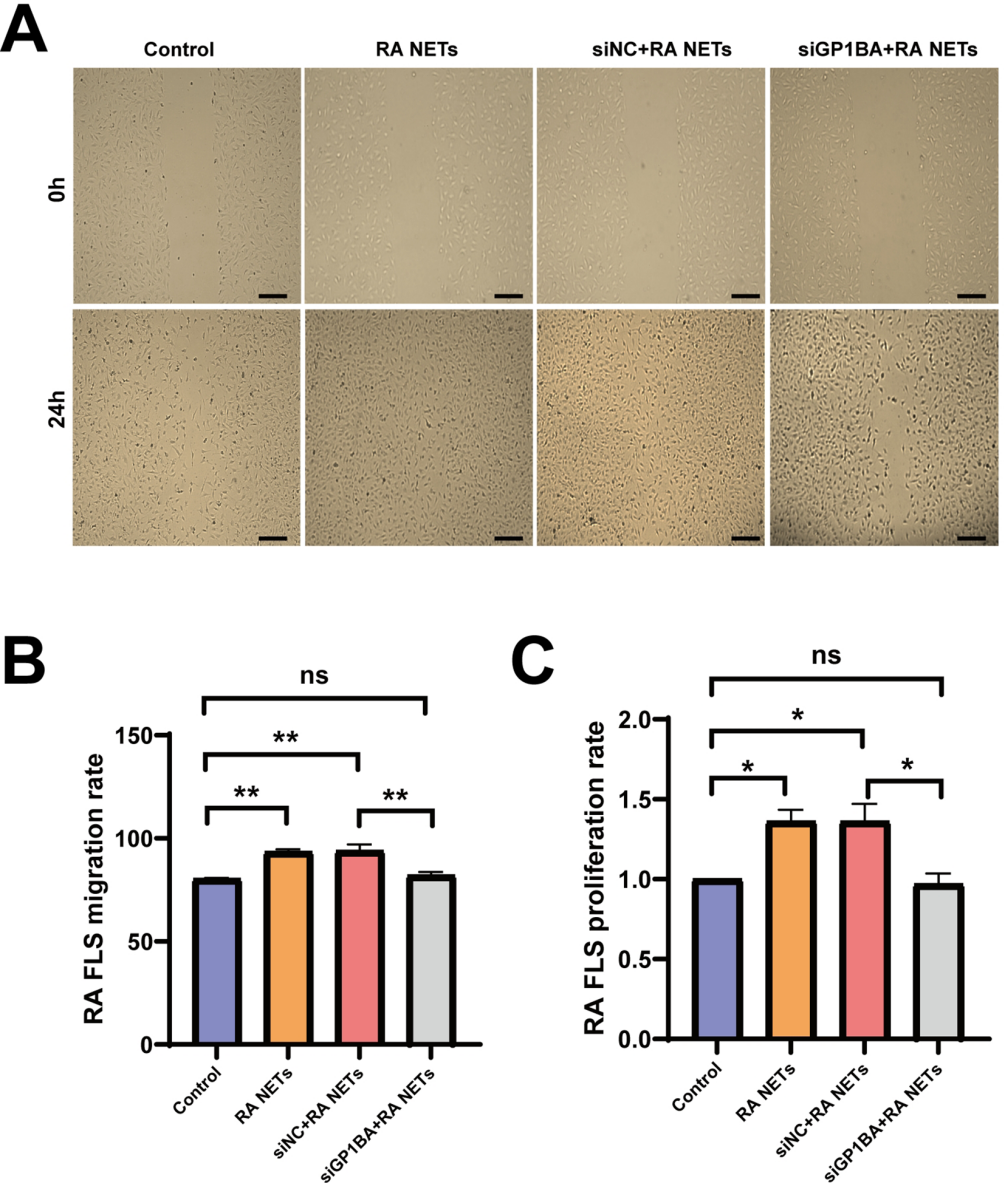
RA NETs promoted RA FLS proliferation and migration via GP1BA. **(A)** Representative images of cell migration in GP1BA-knockdown FLS treated with RA NETs at baseline (0h) and after 24 h (scale bar: 100μm). **(B)** Quantification of migration rates in GP1BA-knockdown FLS stimulated by RA NETs. **(C)** Quantification of proliferation rates in GP1BA-knockdown FLS stimulated by RA NETs. *P < 0.05, **P < 0.01.

To verify the biological function of GP1BA in NETs-mediated FLS invasion, GP1BA was knocked down in RA FLS using siRNA. Our data showed that RA NETs significantly enhanced the migration and proliferation of RA FLS, whereas this effect was largely abolished in GP1BA-silenced cells (**Figure 4**). These findings indicate that GP1BA plays a pivotal regulatory role in RA NETs-mediated FLS invasion.

### PAD4 inhibitor alleviated synovitis in CIA mice

**Figure 5:**
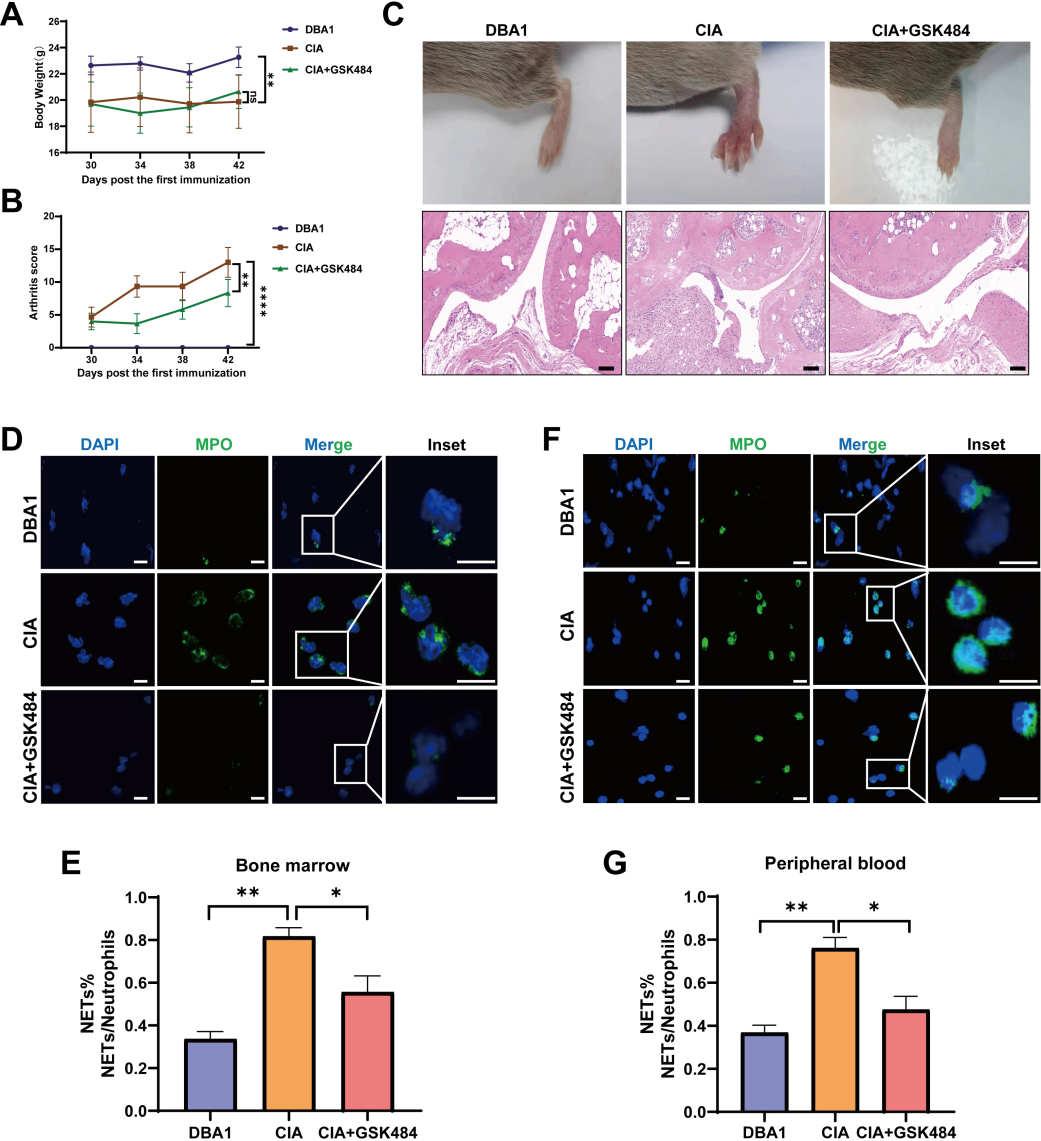
PAD4 inhibitor alleviated synovitis in CIA mice. **(A, B)** Body weight and arthritis scores in different groups (DBA1, CIA and CIA+GSK484) at various time points after CIA immunization. **(C)** Representative H&E-stained sagittal sections of ankle joints from each group, showing inflammation and tissue damage (scale bar: 100μm). **(D, F)** Representative immunofluorescence images of MPO release from neutrophils in bone marrow and peripheral blood (with neutrophil nuclei stained blue, and MPO stained green). Scale bars represent 100μm for the main images and 100μm for the inset images. **(E, G)** Quantification of the percentage of NETs in neutrophils from mouse bone marrow and peripheral blood. *P < 0.05, **P < 0.01.

To further validate the function of NETs *in vivo*, a collagen-induced arthritis (CIA) model was established, and mice were treated with GSK484, a PAD4 inhibitor that blocks NET formation. Compared to CIA mice, GSK484-treated mice exhibited a gradual increase in body weight and a progressive reduction in arthritis scores (**Figures 5A, B**). Histopathological analysis revealed that GSK484 treatment led to a marked reduction in inflammatory cell infiltration within the synovial tissue and significant alleviation of joint damage (**Figure 5C**). Moreover, neutrophils isolated from the peripheral blood and bone marrow of CIA mice released more MPO and formed more NETs than those from healthy DBA/1 mice. This enhanced NETs formation was markedly suppressed by GSK484 treatment (**Figures 5D-G**). Taken together, these findings suggest that excessive NETs formation contributes to synovitis and joint injury in CIA.

### PAD4 inhibitor suppressed FLS invasiveness and GPIbα expression in CIA synovium

**Figure 6:**
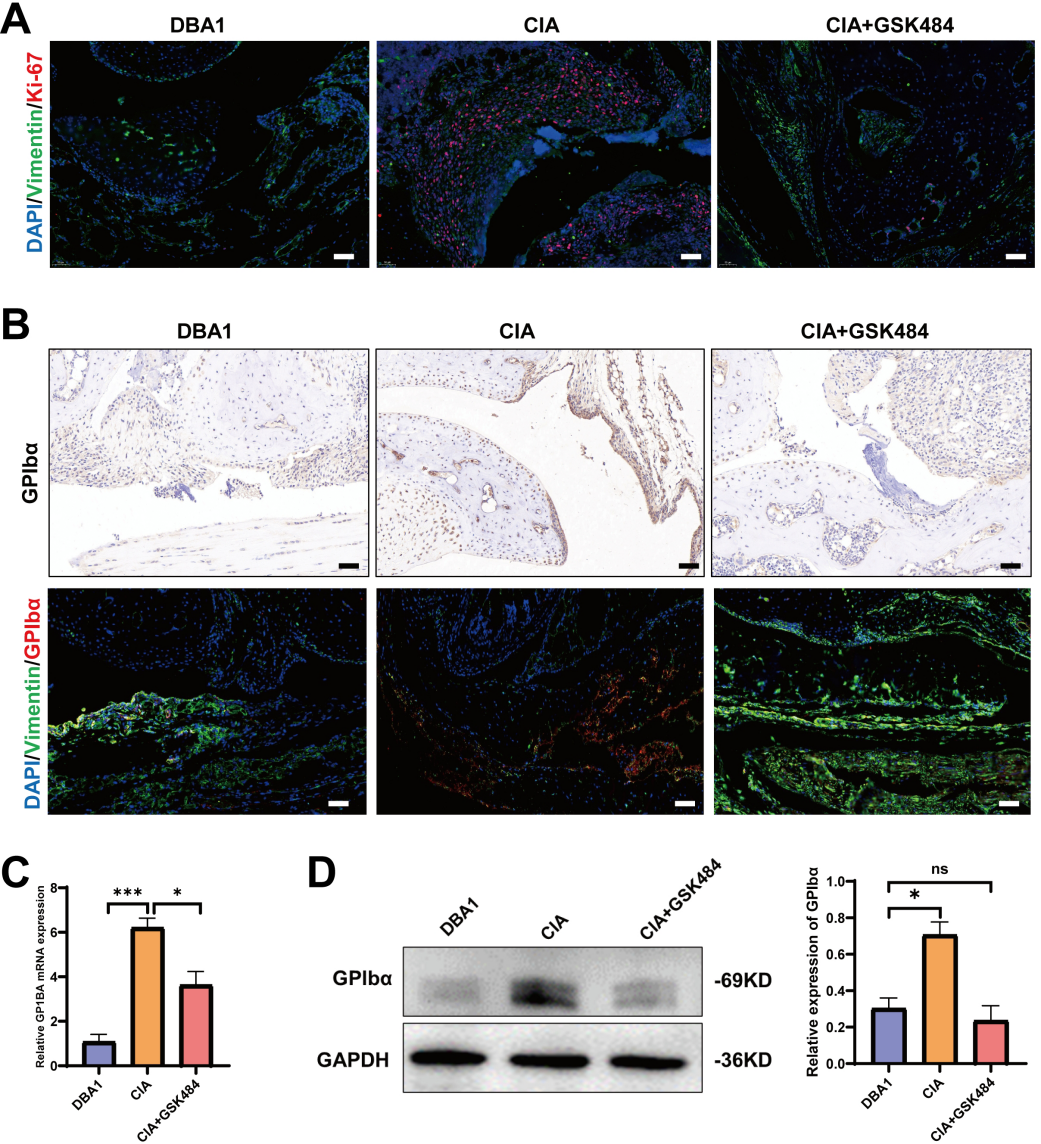
PAD4 inhibitor suppressed FLS invasiveness and GPIbα expression in CIA synovium. **(A)** Representative immunofluorescence images of Ki-67 (red) and vimentin (green) indicating synovial hyperplasia in mouse ankle joint (scale bar: 100μm). DAPI was used to stain the nuclei (blue). **(B)** Immunohistochemical staining of GPIbα (upper panels) and immunofluorescence staining of GPIbα and vimentin (lower panels) in mouse ankle joints (scale bar: 100μm). **(C)** Quantification of *GP1BA* mRNA expression in mouse synovium tissues by RT-qPCR. **(D)** Western blot analysis of GPIbα expression in mouse synovium tissues, with GAPDH as a loading control. *P < 0.05, ***P < 0.001; ns, not significant.

In CIA synovium, Ki-67 expression in Vimentin+ FLS was significantly elevated, while GSK484 treatment markedly reduced Ki-67 levels, confirming that PAD4 inhibitor suppressed FLS invasiveness *in vivo*. (**Figure 6A**). Immunohistochemistry and immunofluorescence staining showed that GPIbα expression was significantly higher in CIA synovium than in healthy DBA/1 mice, and GSK484 treatment effectively reduced its expression, (**Figure 6B**). In addition, RT-qPCR and western blot analyses confirmed that GSK484 downregulated GPIbα at both the mRNA and protein levels in CIA synovium (**Figures 6C, D**). Overall, these findings indicate that NETs inhibition in CIA could alleviate FLS invasive behavior accompanied with downregulation of GPIbα.

## Discussion

RA is characterized by synovitis and abnormal proliferation of synovial cells, with neutrophil infiltration representing a hallmark of early disease pathology (17, 42). In recent years, NETs have emerged as pivotal contributors to RA pathogenesis (43). While previous studies have largely focused on the role of NETs in autoimmunity and citrullinated antigen exposure, their direct influence on FLS remains less explored. This study demonstrates that NETs formation is significantly elevated in RA patients and can promote FLS proliferation and migration by upregulating GPIbα. Importantly, inhibition of NETs formation via a PAD4 inhibitor markedly downregulated GPIbα expression and alleviated disease progression in CIA, suggesting that NETs may not merely be byproducts of the inflammatory microenvironment in RA but active drivers of synovial pathology.

The pathogenesis of RA involves a complex interplay between innate and adaptive immune activation, coupled with aggressive synovial hyperplasia (44, 45). Among synovial cells, FLS are now recognized as central effector cells that not only sustain inflammation but also directly mediate joint destruction (46). Recent evidence indicates that FLS can engage in direct crosstalk with neutrophils, leading to pathogenic autoimmunity and cartilage damage (47).

NETs are web-like structures released by neutrophils in response to various stimuli, primarily functioning to trap and eliminate pathogens. Notably, recent studies have increasingly highlighted their role beyond antimicrobial immunity, particularly in autoimmune diseases such as systemic lupus erythematosus (SLE) and RA (48–50). For instance, excessive NETs formation has been linked to disease severity in SLE, where the LL-37-DNA complex within NETs structures activates TLR9-mediated B cell responses, leading to polyclonal B cell activation, increased antibody production, and exacerbation of inflammation in SLE patients (51). In RA, NETs serve as an important source of citrullinated and other post-translationally modified antigens, which may trigger autoimmune response (52, 53). While these mechanisms highlight the immunogenic role of NETs, our study reveals a more direct pathway: NETs enhance the intrinsic invasiveness of FLS. This finding positions NETs as a bridge between innate immune activation and stromal-driven joint destruction.

Transcriptomic analysis identified *GP1BA* as a key upregulated gene in NETs-stimulated FLS. GPIbα, the core protein encoded by *GP1BA*, is a key receptor mediating platelet adhesion and activation (54), however, its role has been largely overlooked in the context of RA. We provide the first evidence that GPIbα is highly expressed in RA synovial tissues compared to OA controls. Emerging evidence from cancer biology reveals that GPIbα-mediated platelet activation promotes tumor metastasis (55, 56), establishing a paradigm for its function in facilitating cellular invasion. In our study, we observed a similar pro-invasive mechanism in the RA synovium, where NETs-induced GPIbα upregulation in RA FLS significantly enhanced their proliferative and migratory capacities. The molecular mechanisms underlying GPIbα-mediated invasion may involve its ability to reorganize the actin cytoskeleton and activate integrin signaling pathways, as demonstrated in osteosarcoma (55).

*In vivo* results showed the PAD4 inhibitor GSK484 not only reduced NETs formation but also suppressed GPIbα expression and FLS proliferation. These findings align with those of Papadaki et al., who demonstrated that blocking NETs formation attenuates dendritic cell (DC)-mediated Th1 immune responses in RA (57), and further suggest that targeting NETs may simultaneously mitigate both inflammatory and stromal components of the disease. Our mechanistic findings further nominate GPIbα as a potential therapeutic target in RA. Current biologic and synthetic DMARDs primarily modulate immune cell activation or cytokine signaling, yet many patients exhibit incomplete responses or loss of efficacy over time. The NETs-GPIbα axis represents a novel stromal-innate immune interface that could be leveraged to complement existing strategies. Unlike current PAD4 inhibitors, which broadly affect NETs and other PAD4-dependent processes, directly targeting the GPIbα could more specifically disrupt the pro-invasive synovial phenotype while minimizing systemic effects.

However, several limitations of our study warrant consideration. First, the *in vivo* effects of GSK484, though promising, may not be exclusively attributable to NETs inhibition, as PAD4 is involved in other cellular processes. Second, the signaling pathways linking NETs to GPIbα upregulation remain unclear and require further investigation. Finally, while GPIbα appears to be a central mediator, the contribution of other NETs-induced genes cannot be ruled out.

## Conclusion

This study establishes a direct pathogenic link between NETs and FLS behavior in RA, through the upregulation of GPIbα. We show that RA-derived NETs enhance FLS proliferation and migration, and identify GPIbα as a novel, NETs-regulated effector of synovial invasion. In the CIA model, NETs inhibition reduced both GPIbα expression and synovitis, underscoring the translational potential of this pathway. Together, these findings reveal a previously unrecognized mechanism by which neutrophils drive stromal pathology in RA, and highlight GPIbα as a promising target for future therapeutic development.

## Data Availability

The datasets presented in this study can be found in online repositories. The names of the repository/repositories and accession number(s) can be found below: PRJNA1303362 (SRA).

## Supplementary material

Supplementary Figure 1 RA NETs promote the migration of osteoarthritis fibroblast-like synoviocytes (OA FLS). (A) Representative images of the wound healing assay showing OA FLS migration at 0 and 24 hours after stimulation with or without RA NETs (50μg/ml). (B) Quantification of the OA FLS migration rate after 24-hour stimulation with or without RA NETs (50μg/ml).

Supplementary Figure 2 RA FLS exhibit a heightened inflammatory response compared to OA FLS following RA NETs stimulation. (A-D) Relative mRNA expression levels of pro-inflammatory cytokines in RA FLS and OA FLS after 24-hour stimulation with RA NETs (50μg/ml), as determined by RT-qPCR. (E-L) RT-qPCR analysis of the indicated cytokine expression in OA FLS (E-H) and RA FLS (I-L) with or without RA NETs stimulation (50μg/ml for 24 hours).

## References

[1] Di Matteo A Bathon JM Emery P . Rheumatoid arthritis. Lancet. (2023) 402:2019–33. doi: 10.1016/S0140-6736(23)01525-8, PMID: 38240831

[2] Jang S Kwon EJ Lee JJ . Rheumatoid arthritis: pathogenic roles of diverse immune cells. Int J Mol Sci. (2022) 23:905. doi: 10.3390/ijms23020905, PMID: 35055087 PMC8780115

[3] Venetsanopoulou AI Alamanos Y Voulgari PV Drosos AA . Epidemiology of rheumatoid arthritis: genetic and environmental influences. Expert Rev Clin Immunol. (2022) 18:923–31. doi: 10.1080/1744666X.2022.2106970, PMID: 35904251

[4] Finckh A Gilbert B Hodkinson B Bae SC Thomas R Deane KD . Global epidemiology of rheumatoid arthritis. Nat Rev Rheumatol. (2022) 18:591–602. doi: 10.1038/s41584-022-00827-y, PMID: 36068354

[5] Brown P Pratt AG Hyrich KL . Therapeutic advances in rheumatoid arthritis. BMJ. (2024) 384:e070856. doi: 10.1136/bmj-2022-070856, PMID: 38233032

[6] Pap T Dankbar B Wehmeyer C Korb-Pap A Sherwood J . Synovial fibroblasts and articular tissue remodelling: Role and mechanisms. Semin Cell Dev Biol. (2020) 101:140–5. doi: 10.1016/j.semcdb.2019.12.006, PMID: 31956018

[7] Weyand CM Goronzy JJ . The immunology of rheumatoid arthritis. Nat Immunol. (2021) 22:10–8. doi: 10.1038/s41590-020-00816-x, PMID: 33257900 PMC8557973

[8] Kugler M Dellinger M Kartnig F Muller L Preglej T Heinz LX . Cytokine-directed cellular cross-talk imprints synovial pathotypes in rheumatoid arthritis. Ann Rheum Dis. (2023) 82:1142–52. doi: 10.1136/ard-2022-223396, PMID: 37344156

[9] Lotz M Guerne PA . Interleukin-6 induces the synthesis of tissue inhibitor of metalloproteinases-1/erythroid potentiating activity (TIMP-1/EPA). J Biol Chem. (1991) 266:2017–20. doi: 10.1016/S0021-9258(18)52202-X, PMID: 1846608

[10] Bustamante MF Garcia-Carbonell R Whisenant KD Guma M . Fibroblast-like synoviocyte metabolism in the pathogenesis of rheumatoid arthritis. Arthritis Res Ther. (2017) 19:110. doi: 10.1186/s13075-017-1303-3, PMID: 28569176 PMC5452638

[11] Bottini N Firestein GS . Duality of fibroblast-like synoviocytes in RA: passive responders and imprinted aggressors. Nat Rev Rheumatol. (2013) 9:24–33. doi: 10.1038/nrrheum.2012.190, PMID: 23147896 PMC3970924

[12] Sabeh F Fox D Weiss SJ . Membrane-type I matrix metalloproteinase-dependent regulation of rheumatoid arthritis synoviocyte function. J Immunol. (2010) 184:6396–406. doi: 10.4049/jimmunol.0904068, PMID: 20483788

[13] Burn GL Foti A Marsman G Patel DF Zychlinsky A . The neutrophil. Immunity. (2021) 54:1377–91. doi: 10.1016/j.immuni.2021.06.006, PMID: 34260886

[14] Herro R Grimes HL . The diverse roles of neutrophils from protection to pathogenesis. Nat Immunol. (2024) 25:2209–19. doi: 10.1038/s41590-024-02006-5, PMID: 39567761

[15] Zhang L Yuan Y Xu Q Jiang Z Chu CQ . Contribution of neutrophils in the pathogenesis of rheumatoid arthritis. J BioMed Res. (2019) 34:86–93. doi: 10.7555/JBR.33.20190075, PMID: 32305962 PMC7183296

[16] Liu X Li J Sun L Wang T Liang W . The association between neutrophil-to-lymphocyte ratio and disease activity in rheumatoid arthritis. Inflammopharmacology. (2023) 31:2237–44. doi: 10.1007/s10787-023-01273-2, PMID: 37418101

[17] Wright HL Lyon M Chapman EA Moots RJ Edwards SW . Rheumatoid arthritis synovial fluid neutrophils drive inflammation through production of chemokines, reactive oxygen species, and neutrophil extracellular traps. Front Immunol. (2020) 11:584116. doi: 10.3389/fimmu.2020.584116, PMID: 33469455 PMC7813679

[18] Wright HL Moots RJ Edwards SW . The multifactorial role of neutrophils in rheumatoid arthritis. Nat Rev Rheumatol. (2014) 10:593–601. doi: 10.1038/nrrheum.2014.80, PMID: 24914698

[19] Chen J Cao Y Xiao J Hong Y Zhu Y . The emerging role of neutrophil extracellular traps in the progression of rheumatoid arthritis. Front Immunol. (2024) 15:1438272. doi: 10.3389/fimmu.2024.1438272, PMID: 39221253 PMC11361965

[20] Chang Y Ou Q Zhou X Nie K Liu J Zhang S . Global research trends and focus on the link between rheumatoid arthritis and neutrophil extracellular traps: a bibliometric analysis from 1985 to 2023. Front Immunol. (2023) 14:1205445. doi: 10.3389/fimmu.2023.1205445, PMID: 37680637 PMC10481536

[21] Brinkmann V Zychlinsky A . Beneficial suicide: why neutrophils die to make NETs. Nat Rev Microbiol. (2007) 5:577–82. doi: 10.1038/nrmicro1710, PMID: 17632569

[22] Fuchs TA Abed U Goosmann C Hurwitz R Schulze I Wahn V . Novel cell death program leads to neutrophil extracellular traps. J Cell Biol. (2007) 176:231–41. doi: 10.1083/jcb.200606027, PMID: 17210947 PMC2063942

[23] Brinkmann V Zychlinsky A . Neutrophil extracellular traps: is immunity the second function of chromatin? J Cell Biol. (2012) 198:773–83. doi: 10.1083/jcb.201203170, PMID: 22945932 PMC3432757

[24] Brinkmann V Reichard U Goosmann C Fauler B Uhlemann Y Weiss DS . Neutrophil extracellular traps kill bacteria. Science. (2004) 303:1532–5. doi: 10.1126/science.1092385, PMID: 15001782

[25] Merza M Hartman H Rahman M Hwaiz R Zhang E Renstrom E . Neutrophil extracellular traps induce trypsin activation, inflammation, and tissue damage in mice with severe acute pancreatitis. Gastroenterology. (2015) 149:1920–1931.e1928. doi: 10.1053/j.gastro.2015.08.026, PMID: 26302488

[26] Wang Y Du C Zhang Y Zhu L . Composition and function of neutrophil extracellular traps. Biomolecules. (2024) 14:416. doi: 10.3390/biom14040416, PMID: 38672433 PMC11048602

[27] Wigerblad G Kaplan MJ . Neutrophil extracellular traps in systemic autoimmune and autoinflammatory diseases. Nat Rev Immunol. (2023) 23:274–88. doi: 10.1038/s41577-022-00787-0, PMID: 36257987 PMC9579530

[28] Aymonnier K Amsler J Lamprecht P Salama A Witko-Sarsat V . The neutrophil: A key resourceful agent in immune-mediated vasculitis. Immunol Rev. (2023) 314:326–35. doi: 10.1111/imr.13170, PMID: 36408947

[29] Herster F Bittner Z Archer NK Dickhöfer S Eisel D Eigenbrod T . Neutrophil extracellular trap-associated RNA and LL37 enable self-amplifying inflammation in psoriasis. Nat Commun. (2020) 11:105. doi: 10.1038/s41467-019-13756-4, PMID: 31913271 PMC6949246

[30] O'Neil LJ Oliveira CB Wang X Navarrete M Barrera-Vargas A Merayo-Chalico J . Neutrophil extracellular trap-associated carbamylation and histones trigger osteoclast formation in rheumatoid arthritis. Ann Rheum Dis. (2023) 82:630–8. doi: 10.1136/ard-2022-223568, PMID: 36737106 PMC11302494

[31] Schneider AH Taira TM Publio GA Da Silva Prado D Donate Yabuta PB Dos Santos JC . Neutrophil extracellular traps mediate bone erosion in rheumatoid arthritis by enhancing RANKL-induced osteoclastogenesis. Br J Pharmacol. (2024) 181:429–46. doi: 10.1111/bph.16227, PMID: 37625900

[32] Aletaha D Neogi T Silman AJ Funovits J Felson DT Bingham CO 3rd . Rheumatoid arthritis classification criteria: an American College of Rheumatology/European League Against Rheumatism collaborative initiative. Arthritis Rheum. (2010) 62:2569–81. doi: 10.1002/art.27584, PMID: 20872595

[33] Arnett FC Edworthy SM Bloch DA McShane DJ Fries JF Cooper NS . The American Rheumatism Association 1987 revised criteria for the classification of rheumatoid arthritis. Arthritis Rheum. (1988) 31:315–24. doi: 10.1002/art.1780310302, PMID: 3358796

[34] Bessis N Decker P Assier E Semerano L Boissier MC . Arthritis models: usefulness and interpretation. Semin Immunopathol. (2017) 39:469–86. doi: 10.1007/s00281-017-0622-4, PMID: 28349194

[35] Sun Y Kong W Huang S Shi B Zhang H Chen W . Comparable therapeutic potential of umbilical cord mesenchymal stem cells in collagen-induced arthritis to TNF inhibitor or anti-CD20 treatment. Clin Exp Rheumatol. (2017) 35:288–95. 28094754

[36] Fukuchi M Miyabe Y Furutani C Saga T Moritoki Y Yamada T . How to detect eosinophil ETosis (EETosis) and extracellular traps. Allergol Int. (2021) 70:19–29. doi: 10.1016/j.alit.2020.10.002, PMID: 33189567 PMC9333458

[37] Schoenfeld L Appl B Pagerols-Raluy L Heuer A Reinshagen K Boettcher M . Immunofluorescence imaging of neutrophil extracellular traps in human and mouse tissues. J Vis Exp. (2023) 198:e65272. doi: 10.3791/65272, PMID: 37677039

[38] Zhang YQ Ding CZ Sun Y . Sinomenine inhibits PDGF/PDGFR signaling pathway to reduce RA FLS migration induced by NETs. Zhongguo Zhong Yao Za Zhi. (2024) 49:1947–55. doi: 10.19540/j.cnki.cjcmm.20240110.502, PMID: 38812207

[39] Li R . Recent advances on GPIb-IX-V complex. Platelets. (2022) 33:809–10. doi: 10.1080/09537104.2022.2075146, PMID: 35543611 PMC9378636

[40] Yuan L Xu H Guo R Lu T Li X . Long non-coding RNA ZFAS1 alleviates bupivacaine-induced neurotoxicity by regulating the miR-421/zinc finger protein564 (ZNF564) axis. Bioengineered. (2021) 12:5231–40. doi: 10.1080/21655979.2021.1960776, PMID: 34414857 PMC8806570

[41] Liessmann F Von Bredow L Meiler J Liebscher I . Targeting adhesion G protein-coupled receptors. Current status and future perspectives. Structure. (2024) 32:2188–205. doi: 10.1016/j.str.2024.10.022, PMID: 39520987

[42] Cheng L Wang Y Wu R Ding T Xue H Gao C . New insights from single-cell sequencing data: synovial fibroblasts and synovial macrophages in rheumatoid arthritis. Front Immunol. (2021) 12:709178. doi: 10.3389/fimmu.2021.709178, PMID: 34349767 PMC8326910

[43] Woodman I . Rheumatoid arthritis: unravelling the roles of NETs in RA. Nat Rev Rheumatol. (2013) 9:258. doi: 10.1038/nrrheum.2013.59, PMID: 23591486

[44] Edilova MI Akram A Abdul-Sater AA . Innate immunity drives pathogenesis of rheumatoid arthritis. BioMed J. (2021) 44:172–82. doi: 10.1016/j.bj.2020.06.010, PMID: 32798211 PMC8178572

[45] D'Orazio A Cirillo AL Greco G Di Ruscio E Latorre M Pisani F . Pathogenesis of rheumatoid arthritis: one year in review 2024. Clin Exp Rheumatol. (2024) 42:1707–13. doi: 10.55563/clinexprheumatol/0307ed, PMID: 39315569

[46] Bartok B Firestein GS . Fibroblast-like synoviocytes: key effector cells in rheumatoid arthritis. Immunol Rev. (2010) 233:233–55. doi: 10.1111/j.0105-2896.2009.00859.x, PMID: 20193003 PMC2913689

[47] Carmona-Rivera C Carlucci PM Moore E Lingampalli N Uchtenhagen H James E . Synovial fibroblast-neutrophil interactions promote pathogenic adaptive immunity in rheumatoid arthritis. Sci Immunol. (2017) 2:eaag3358. doi: 10.1126/sciimmunol.aag3358, PMID: 28649674 PMC5479641

[48] Fousert E Toes R Desai J . Neutrophil extracellular traps (NETs) take the central stage in driving autoimmune responses. Cells. (2020) 9:915. doi: 10.3390/cells9040915, PMID: 32276504 PMC7226846

[49] Fresneda Alarcon M McLaren Z Wright HL . Neutrophils in the pathogenesis of rheumatoid arthritis and systemic lupus erythematosus: same foe different M.O. Front Immunol. (2021) 12:649693. doi: 10.3389/fimmu.2021.649693, PMID: 33746988 PMC7969658

[50] Lee KH Kronbichler A Park DD Park Y Moon H Kim H . Neutrophil extracellular traps (NETs) in autoimmune diseases: A comprehensive review. Autoimmun Rev. (2017) 16:1160–73. doi: 10.1016/j.autrev.2017.09.012, PMID: 28899799

[51] Gestermann N Di Domizio J Lande R Demaria O Frasca L Feldmeyer L . Netting neutrophils activate autoreactive B cells in lupus. J Immunol. (2018) 200:3364–71. doi: 10.4049/jimmunol.1700778, PMID: 29632142

[52] Corsiero E Pratesi F Prediletto E Bombardieri M Migliorini P . NETosis as source of autoantigens in rheumatoid arthritis. Front Immunol. (2016) 7:485. doi: 10.3389/fimmu.2016.00485, PMID: 27895639 PMC5108063

[53] Wu S Peng W Liang X Wang W . Anti-citrullinated protein antibodies are associated with neutrophil extracellular trap formation in rheumatoid arthritis. J Clin Lab Anal. (2021) 35:e23662. doi: 10.1002/jcla.23662, PMID: 33249645 PMC7957993

[54] Huang L Shao B . New insights of glycoprotein Ib-IX-V complex organization and glycoprotein Ibalpha in platelet biogenesis. Curr Opin Hematol. (2024) 31:294–301. doi: 10.1097/MOH.0000000000000832, PMID: 39046849 PMC12532385

[55] Wang Q Liu W Fan J Guo J Shen F Ma Z . von Willebrand factor promotes platelet-induced metastasis of osteosarcoma through activation of the VWF-GPIb axis. J Bone Oncol. (2020) 25:100325. doi: 10.1016/j.jbo.2020.100325, PMID: 33101888 PMC7569326

[56] Jain S Zuka M Liu J Russell S Dent J Guerrero JA . Platelet glycoprotein Ib alpha supports experimental lung metastasis. Proc Natl Acad Sci U.S.A. (2007) 104:9024–8. doi: 10.1073/pnas.0700625104, PMID: 17494758 PMC1885621

[57] Papadaki G Kambas K Choulaki C Vlachou K Drakos E Bertsias G . Neutrophil extracellular traps exacerbate Th1-mediated autoimmune responses in rheumatoid arthritis by promoting DC maturation. Eur J Immunol. (2016) 46:2542–54. doi: 10.1002/eji.201646542, PMID: 27585946 PMC5476297

