# Supplementary figures and images for: NETs promote invasive behavior of fibroblast-like synoviocytes through GPIbα in rheumatoid arthritis
Source: Front Immunol. 2025 Nov 21;16:1667319. doi: 10.3389/fimmu.2025.1667319 (PMC12678396; doi:10.3389/fimmu.2025.1667319)

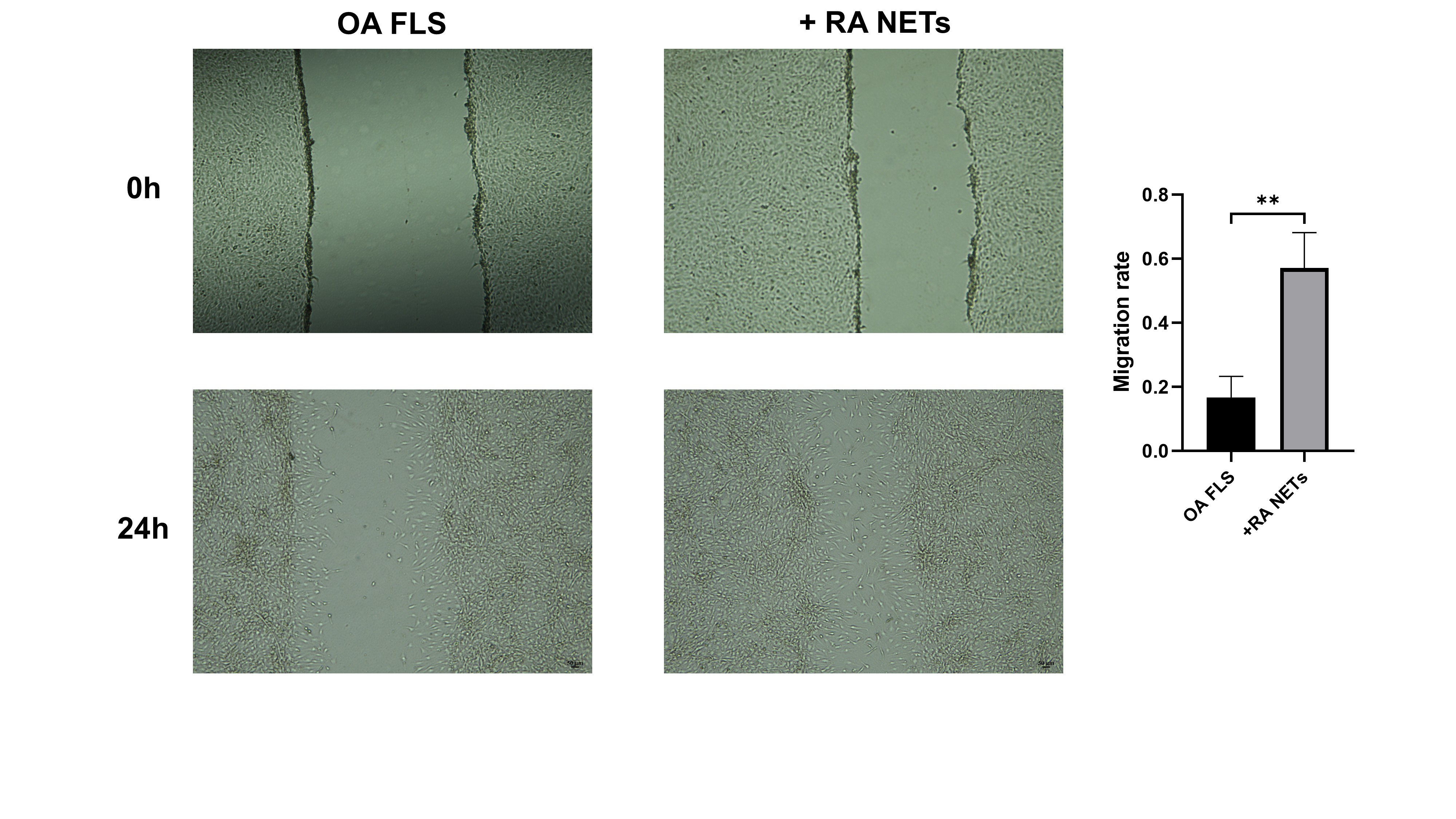

Supplement: Supplementary Figure 1 — RA NETs promote the migration of osteoarthritis fibroblast-like synoviocytes (OA FLS). (A) Representative images of the wound healing assay showing OA FLS migration at 0 and 24 hours after stimulation with or without RA NETs (50μg/ml). (B) Quantification of the OA FLS migration rate after 24-hour stimulation with or without RA NETs (50μg/ml). [file Image1.jpg]

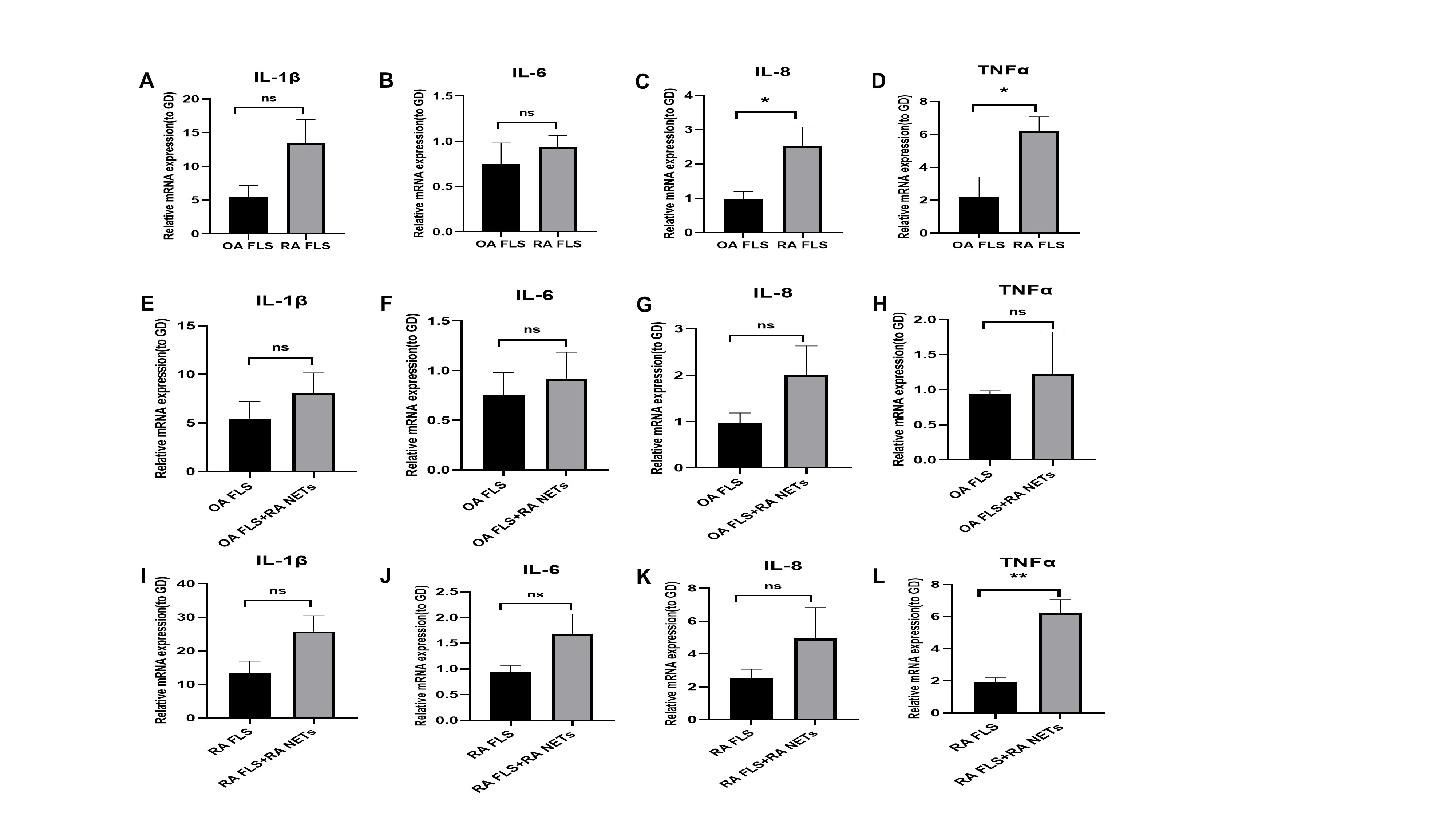

Supplement: Supplementary Figure 2 — RA FLS exhibit a heightened inflammatory response compared to OA FLS following RA NETs stimulation. (A-D) Relative mRNA expression levels of pro-inflammatory cytokines in RA FLS and OA FLS after 24-hour stimulation with RA NETs (50μg/ml), as determined by RT-qPCR. (E-L) RT-qPCR analysis of the indicated cytokine expression in OA FLS (E-H) and RA FLS (I-L) with or without RA NETs stimulation (50μg/ml for 24 hours). [file Image2.jpg]
